# Cruciferous vegetable supplementation in a controlled diet study alters the serum peptidome in a *GSTM1*-genotype dependent manner

**DOI:** 10.1186/1475-2891-10-11

**Published:** 2011-01-27

**Authors:** Heather Ann Brauer, Tanya E Libby, Breeana L Mitchell, Lin Li, Chu Chen, Timothy W Randolph, Yutaka Y Yasui, Johanna W Lampe, Paul D Lampe

**Affiliations:** 1 Molecular and Cellular Biology Program, University of Washington, Seattle, WA 98195, USA; 2 Fred Hutchinson Cancer Research Center, Seattle, WA 98109, USA; 3 Department of Otolaryngology: Head and Neck Surgery, University of Washington, Seattle, WA 98195, USA; 4 Department of Epidemiology, University of Washington, Seattle, WA 98195, USA; 5 Department of Public Health Sciences, University of Alberta, Edmonton, AB, T6G 2G3, Canada

## Abstract

**Background:**

Cruciferous vegetable intake is inversely associated with the risk of several cancers. Isothiocyanates (ITC) are hypothesized to be the major bioactive constituents contributing to these cancer-preventive effects. The polymorphic glutathione-*S*-transferase (GST) gene family encodes several enzymes which catalyze ITC degradation *in vivo*.

**Methods:**

We utilized high throughput proteomics methods to examine how human serum peptides (the "peptidome") change in response to cruciferous vegetable feeding in individuals of different *GSTM1 *genotypes. In two randomized, crossover, controlled feeding studies (EAT and 2EAT) participants consumed a fruit- and vegetable-free basal diet and the basal diet supplemented with cruciferous vegetables. Serum samples collected at the end of the feeding period were fractionated and matrix assisted laser desorption/ionization-time of flight (MALDI-TOF) mass spectrometry spectra were obtained. Peak identification/alignment computer algorithms and mixed effects models were used to analyze the data.

**Results:**

After analysis of spectra from EAT participants, 24 distinct peaks showed statistically significant differences associated with cruciferous vegetable intake. Twenty of these peaks were driven by their *GSTM1 *genotype (i.e., *GSTM1+ *or *GSTM1- *null). When data from EAT and 2EAT participants were compared by joint processing of spectra to align a common set, 6 peaks showed consistent changes in both studies in a genotype-dependent manner. The peaks at 6700 *m/z *and 9565 *m/z *were identified as an isoform of transthyretin (TTR) and a fragment of zinc α2-glycoprotein (ZAG), respectively.

**Conclusions:**

Cruciferous vegetable intake in *GSTM1+ *individuals led to changes in circulating levels of several peptides/proteins, including TTR and a fragment of ZAG. TTR is a known marker of nutritional status and ZAG is an adipokine that plays a role in lipid mobilization. The results of this study present evidence that the *GSTM1*-genotype modulates the physiological response to cruciferous vegetable intake.

## Background

Intake of vegetables from the family Brassicaceae (i.e., cruciferous or broccoli-family) is inversely associated with the risk of several cancers [1]. The influence of cruciferous vegetable intake on cancer risk has been hypothesized to be through several mechanisms, including reduction in systemic oxidative stress [2], suppression of mutagenic and carcinogenic activity [3,4], reduction in proliferation [5,6] and induction of phase II detoxifying enzymes, such as glutathione *S*-transferases (GST) and UDP-glucuronosyltransferases (UGT) [7,8]. Glucosinolates in cruciferous vegetables that can be hydrolyzed to yield isothiocyanates (ITC) and indole-3-carbinol are hypothesized to be the major bioactive constituents contributing to these cancer-preventive effects.

ITC are metabolized through the mercapturic acid pathway, with GST conjugation of ITC and glutathione as the first step in the pathway toward degradation [9]. Thus, ITC are substrates for several GST enzymes, particularly *GSTM1*[10]. A homozygous deletion of the *GSTM1 *gene results in no detectable *GSTM1* activity. Hence, *GSTM1*-null individuals may have greater exposure to ITC [11], although pharmacokinetic studies do not conclusively support this concept [12,13]. It is possible that other GST isozymes may partially compensate for the lack of *GSTM1*[14].

Previously, we showed that matrix assisted laser desorption/ionization-time of flight (MALDI-TOF) analysis of serum proteins could distinguish individuals on the basis of whether they were consuming controlled diets devoid of, or supplemented with, cruciferous vegetables; two specific peptide markers, one of which was the B-chain of alpha 2-HS glycoprotein (AHSG B-chain), had the ability to discriminate most accurately between diets [15]. Here we analyze samples from a more recent and comprehensive controlled feeding study [8] with the objectives to: 1) confirm the previous results, 2) evaluate the dose-response effect of cruciferous vegetables on the serum peptidome in a separate, independent sample, and 3) examine the peptidomic response to diet in the context of *GSTM1+ *genotype. We hypothesized that, due to the potential differences in ITC metabolism between *GSTM1*+ and *GSTM1-null *individuals, the serum peptidome would differ by genotype. Our results indicate that *GSTM1 *genotype differences affect levels of particular peptides/proteins in a sensitive and specific manner.

## Methods

### Research Design

The analyses were conducted using stored serum samples collected as part of two completed studies, "The Enzyme Activation Trial" (EAT) [16] and "Enzyme Activation Trial 2" (2EAT) [8], conducted at the Fred Hutchinson Cancer Research Center (FHCRC), Seattle, Washington. These were randomized, crossover, controlled feeding studies designed to test the effects of vegetable diets and *GST *genotypes on biotransformation enzyme activity and other biomarkers of cancer susceptibility. In both studies, participants were recruited and block randomized to diet order based on *GST *genotype and sex [8].

### Study Participants

In the EAT study, participants were healthy, non-smokers, aged 20-40 years, and were recruited on the basis of sex and *GSTM1 *genotypes. For the current ancillary study, analyses were conducted on 36 individuals, a subset of the 40 individuals who completed all 4 study periods and for whom we had sample available: 17 women (4 *GSTM1*+ and 13 *GSTM1-null*) and 19 men (10 *GSTM1*+ and 9 *GSTM1-null*) [16] (Table 1).

**Table 1:** Gender and genotype distribution of participants for EAT and 2EAT^1^

	Men		Women	
	***GSTM1+ ****(n)*	***GSTM1- ****(n)*	***GSTM1+ ****(n)*	***GSTM1- ****(n)*

**EAT Study**	10	9	4	13

**2EAT Study**	10	15	10	7

^1^*GSTM1 *genotypes (i.e., *GSTM1+ *or *GSTM1- *null) by gender are presented for both dietary studies; all participants were used for analysis.

In the 2EAT study, participants were healthy, non-smokers, aged 20-40 years, and were recruited on the basis of sex and *CYP1A2 *and *GSTM1 *genotypes as described previously [8]. In all, 72 individuals ultimately were randomized into the parent study; however, for our current analysis, only samples and data from the first 42 individuals were included. The distribution of genotype among these 42 individuals was as follows: 17 women (10 *GSTM1*+ and 7 *GSTM1-null*) and 25 men (10 *GSTM1*+ and 15 *GSTM1-null*) (Table 1). The Institutional Review Board at the FHCRC approved the studies and informed written consent was obtained from all participants prior to the start of these studies.

### Study Diets

The EAT and 2EAT studies were conducted as randomized, controlled feeding studies. Study diets were prepared by the FHCRC Human Nutrition Lab. Dinner was served in the Human Nutrition Lab dining room Sunday through Friday evening, and food for the following day's morning, midday meal, and snacks was distributed at that time. On Friday evening, participants picked up food for Saturday and Sunday meals. The major portion of the test vegetables was provided as part of the dinner under the supervision of the study staff. Participants were instructed to consume only the food and beverages provided for them during the diet periods. They were requested not to take any medication, alcoholic beverages, or nutritional supplements and to maintain their usual physical activity during each period. Any food not consumed was recorded on the participant's chart. Compliance with the study diet and deviations from the diet were monitored using daily food check-off forms.

In the EAT feeding study, participants consumed four different controlled diets: (i) a basal diet devoid of fruit and vegetables; (ii) the basal diet supplemented with a prescribed amount of cruciferous vegetables (436 g/day of a mixture of broccoli, cabbage, cauliflower and radish sprouts); (iii) the basal diet supplemented with allium vegetables (90 g/day of a mixture of chives, leeks, garlic and onions); (iv) and the basal diet supplemented with apiaceous vegetables (270 g/day of a mixture of carrots, celery, dill weed, parsley and parsnips) [16,17]. Designed originally to test the effects of diet on biotransformation enzymes, each of the four diet periods lasted 6 days with at least a 2 week washout period between diets. Biotransformation enzymes respond rapidly to diet [18] and ITC doses are cleared in urine in 24 hours [12], supporting the lengths of the treatment periods and washouts. In this study, all individuals received the same doses of vegetables [16,17].

In the 2EAT feeding study, participants consumed four different controlled diets: (i) a basal diet devoid of fruit and vegetables; (ii) the basal diet supplemented with a prescribed amount of cruciferous vegetables [single-dose (1X) cruciferous diet, 7 g/kg-body-weight of a mixture of broccoli, cabbage, cauliflower and radish sprouts]; (iii) the basal diet supplemented with twice this amount of cruciferous vegetables [double-dose (2X) cruciferous diet, 14 g/kg body weight]; (iv) and the basal diet supplemented with 7 g cruciferous vegetables/kg body weight and 4 g apiaceous vegetables/kg body weight (1X cruciferous plus apiaceous vegetable diet; the apiaceous vegetables include a mix of carrots, celery, dill weed, parsley and parsnips) [8]. Diet order was randomly assigned in the cross-over design and controlled for in the analysis. Each of the four diet periods lasted 14 days with at least a 21 day washout period between diets and as indicated, vegetables were dosed by body weight [8].

Our initial mass spectrometry analysis indicated that the largest differences were between the cruciferous and basal diets, so the apiaceous and allium vegetable diets (from EAT, diet iii and iv; from 2EAT, diet iv) were not included in further analyses [15].

### Specimen collection

For the EAT study, blood samples obtained on the last day (day 7) of each feeding period were used for this analysis. Blood was drawn in the morning following an 8-12 hour overnight fast. Blood was allowed to clot at room temperature for 30 min before it was centrifuged to separate the serum. Serum was aliquotted and stored at -80°C [16]. For the 2EAT study, blood was collected from participants on days 0, 7, 11 and day 15 of each feeding period; only samples from day 15 of the basal and 2 cruciferous vegetable diets were used for this analysis. Blood was drawn in the morning after a 12 hour, overnight fast and serum obtained, aliquoted and stored as for the EAT study [8,16,17]. *GSTM1 *genotyping was conducted on buccal cell DNA as described previously [8,19].

### Sample Preparation

Highly abundant serum proteins, such as albumin and IgG, can cause crystallization defects and reduce the flight of low abundant proteins and peptides in TOF analyses. Therefore, after evaluating a number of methods, we chose a commonly used method to precipitate large globular proteins by mixing 25 uL serum 1:1 with 100% acetonitrile (AcN), shaking on a Vortex Genie 2 (Scientific Industries, Inc., Bohemia, NY) for 30 min and centrifugation [15]. Supernatant was mixed 1:1 with 4-hydroxy-3,5-dimethoxycinnamic acid (sinapic acid) matrix (Sigma-Aldrich, St. Louis, MO) and 1 μL was spotted onto a hydrophobic 384 well MALDI plate in quintuplicate.

### Mass Spectrometry

Samples were analyzed using MALDI-TOF MS on an Applied Biosystem Voyager DE-Pro spectrometer (Foster City, CA). Daily machine calibrations were performed using cytochrome c [12361 (+1), 6181 (+2)] to obtain mass accuracy. Plates were analyzed in linear mode using a 337 nm nitrogen laser, under the following settings optimized using cytochrome c: laser intensity 1,784; accelerating voltage 25,000; grid voltage 23,000; guide wire voltage 1,250; delay time 325 ns; low mass gate 1,000; molecular mass range 1,000-40,000. Final spectra were generated by summing spectra produced from 20 laser hits at up to 20 places per spot (a total of up to 400 laser hits per summed spectra).

### Peak Identification and Alignment

To allow for direct comparison, the two datasets were aligned and processed to formulate a common set of peaks among spectra using procedures as previously described [20]. The intensity values of the resulting aligned peaks were then transformed and standardized as previously described [21,22].

Briefly, an intensity value, *Y*_*ijk *_for subject *i*, spectrum *j *at peak *k*, was transformed to *T*_*ijk *_= ln(*Y*_*ijk *_- *c*_*k *_+ 0.1) where *c*_*k *_is the minimum intensity measured at the *k*^th ^peak among all spectra. The value 0.1 is arbitrary and included to stabilize the logarithm of any extremely small value; *Y*_*ijk*_-*c*_*k *_was the minimum intensity measured at the *k*^th ^peak among all spectra.

### Statistical Analysis

Transformed peak intensities were used in a linear mixed model that included fixed-effect covariates of sex, *GSTM1 *genotype, feeding period, diet treatment, feeding order and an interaction term between genotype and diet; a random-effects term for 'participant' was included to accommodate the crossover feeding design. The same model was used in both EAT and 2EAT feeding studies. To make these data compatible, it was necessary to jointly process and align spectra from both studies to obtain a list of peaks common to both sets of spectra. The peak intensities at each m/z value in this list were independently estimated by the back-transformed least squares for each study. To find the peaks of interest that were consistent in both EAT and 2EAT and, hence, worthy of further study, we applied the following criterion: (1) the peaks shown to be significant (p < 0.05 from the linear mixed model) when comparing the ratio of basal to 1X cruciferous in EAT and 2EAT or the ratio of basal to 2X cruciferous in 2EAT study; (2) peaks from step 1 that have similar size in both studies for the basal and 1X cruciferous diets (EAT has no 2X diet); (3) peaks from steps 1 and 2 that exhibit the same trend when comparing basal and 1X cruciferous diets in both studies. The peaks selected with these criteria were used for further investigation. All statistical analyses were performed using the SAS Program (version 9.1; SAS Institute). Two-sided *P*-value for statistical significance was set at < 0.05.

To prevent confounding by body weight between the sexes in the second study, the amount of vegetables provided was done on a per-kg body-weight basis, and ranged from 284 g to 662 g for the single-dose cruciferous diet and 568 g to 1324 g for the double-dose cruciferous diet. Analyses were conducted both with and without adjusting for vegetable amount; because there were no statistically significant differences between the analyses with and without adjustment, the data are presented without adjustment.

Analysis of significance for the western and dot blots was done using linear regression of the average transthyretin (TTR) and zinc α2-glycoprotein (ZAG) levels associated with cruciferous dose according to genotype, adjusted for the TTR or ZAG levels from basal feeding period with robust standard error [23]. In addition, generalized estimating equations were used with an exchangeable working correlation structure and averages were regressed of any level of TTR or ZAG from cruciferous intake on genotype and adjusted for basal levels.

### Protein Identification

To identify peptides represented by the statistically significant peaks from the MALDI-TOF analysis, albumin and IgG were removed from the sample via PROT-IA spin columns (Sigma-Aldrich, St. Louis, MO). Laemmli sample buffer was added to the eluate and the sample was heated at 100°C for 5 min. The proteins were separated on a NuPAGE 4-12% Bis-Tris Gel (Invitrogen Corporation, Carlsbad, CA) using 2-(N-morpholino) ethane sulfonic acid (MES) running buffer (Invitrogen). The gel was silver stained and bands corresponding to the correct size were excised. The gel slices were digested with trypsin in 50 mmol/L ammonium bicarbonate overnight at 37°C. Peptides were extracted from the gel slice and desalted with a C18 ZipTip (Millipore, Billerica, MA). The material was then analyzed by nano LC/MS using a ThermoFisher LTQ (Thermo Fisher Scientific, Waltham, MA). Proteins were identified using Xtandem. The database search was performed against the human IPI database using Protein Prophet. Results were uploaded to Computational Portal and Analysis System (CPAS) database and interrogated using the following filters to look for individual proteins or peptides: Ion Percent ≥ 0.15; +1 Raw Score ≥ 200; + 2 Raw Score ≥ 300; + 3 Raw Score ≥ 300; Unique Peptides > 1.

### Fractionation of serum for peptide identification

Control serum (3 mL) was precipitated with an equal volume of AcN. The protein concentration of the supernatant from the precipitation was determined using a BCA Protein Assay (Thermo Fisher Scientific). A total of 4 mg protein was concentrated to 500 uL in a Thermo Scientific SpeedVac and loaded onto a Poros R2/10 RP column (Applied Biosystems) attached to a Shimadzu SCL-10A liquid chromatograph. Chromatography was performed at a flow rate of 2.7 mL/min using aqueous AcN-TFA solvents (buffer A: 5% AcN in 0.1% TFA in water; buffer B: 90% AcN and 0.1% TFA in 10% water). The column was equilibrated at 90% buffer A. Buffer B was increased to 5% over 10 min, then to 60% over the next 57 min, and finally to 95% in the last 6 min. The column eluate was monitored at 215 and 280 nm. Fractions were collected every 30 sec. All fractions were analyzed *via *MALDI-TOF to identify those containing the peptide of interest.

### Immunoblot Analyses

For immunoblot analyses, serum samples from the basal and 2X cruciferous diets for 4 participants in the 2EAT study were chosen based on high signal-to-noise ratios in the range of interest. Each sample (25 μL) was precipitated with 25 μL AcN. The protein concentration of the supernatant from the precipitation was determined using a BCA Protein Assay (Thermo Fisher Scientific). Seventy-five micrograms of protein per sample was boiled for 5 min in 3X sample buffer and separated by sodium dodecyl sulfate-polyacrylamide gel electrophoresis on a 10% gel. Protein was transferred onto nitrocellulose membrane and the membrane was blocked with 5% milk in PBS at room temperature. The blot was incubated overnight with a TTR antibody (Dako, Carpinteria, CA) at 1:500 dilution. Primary antibodies were visualized with a fluorescent-dye-labeled secondary antibody (Alexa Fluor-680 goat anti-rabbit, Molecular Probes, Eugene, OR) and directly quantified using the LI-COR Biosciences Odyssey infrared imaging system (Lincoln, NE) and associated software.

For dot blot analyses, serum samples (1 μL) from each of the diets for 2EAT participants were spotted in a 96-well format onto nitrocellulose and the level of TTR or ZAG was visualized as described above. For ZAG, the blot was incubated overnight with a ZAG antibody (R&D Systems, Minneapolis, MN) at 1:250 dilution.

## Results

### Analysis of diet and genotype in EAT

**Figure 1:**
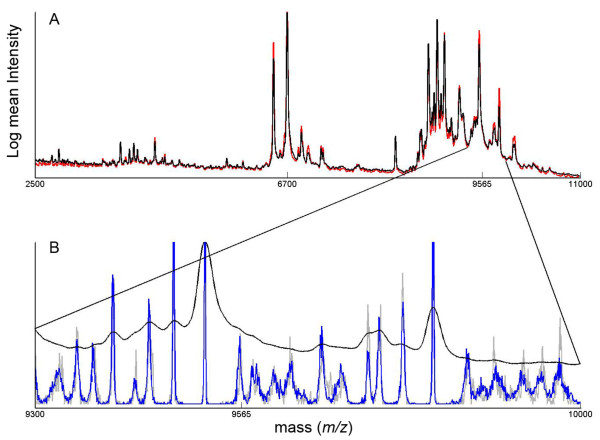
**Processed MALDI-TOF MS spectra. **(A) The 2500-10000 m/z range of the log mean spectrum from each study (all spectra from EAT in red, 2EAT in black) showing the consistency of the data between the two studies and the peak at 7600; (B) A close-up of 2EAT mean spectrum (in black) with peak-location density histograms from all spectra in each study (EAT in gray, 2EAT in blue) shows there are approximately 30 peaks in the 9300-10000 range and the ZAG fragment at 9560 m/z appears consistently on the shoulder of the peak at 9520 m/z.

In the EAT feeding study, participants consumed a basal diet devoid of fruits and vegetables for 6 days, and, in a different feeding period, the basal diet supplemented with cruciferous vegetables (436 g/day of a mixture of broccoli, cabbage, cauliflower and radish sprouts) also for 6 days. MALDI-TOF spectra (1,000-40,000 m/z range) of participant serum were collected for the basal and cruciferous diets for the 36 study participants in EAT. A peak alignment and selection algorithm was applied to the raw spectra and a statistical analysis of the basal diet *versus *the cruciferous diet was performed on the resulting 736 distinct peaks. In Figure 1, we show an example of a processed MALDI-TOF spectrum in the 2,500-10,000 m/z range illustrating peak alignment and density in this range. The analysis accounted for the cross-over study design in which each participant ate both the basal and the cruciferous diet, and identified 24 statistically significant (P < 0.05) peaks that differed between the two diets.

Because variation in GST activity has been hypothesized to influence response to ITC exposure, we investigated the effect of *GSTM1 *genotypes on response to diet. Comparisons of the basal diet to the cruciferous diet within each genotype were performed. There were 21 peaks from *GSTM1-null *and 39 peaks from *GSTM1+ *individuals that changed in a statistically significant manner as a result of cruciferous vegetable intake. Of the original 24 peaks that were significant independent of *GSTM1 *genotype, 20 were still significant in one genotype or the other. Determination of the actual relevance of these peaks to cruciferous diet from these data alone is difficult given that some may appear significant by chance. Samples from a second diet study, 2EAT, were used to look for consistent changes in an independent population to confirm the differences and the *GSTM1 *effect.

### Analysis of diet and genotype in 2EAT

In the 2EAT feeding study, participants consumed the controlled diets for 14 days, and we compared (i) the basal diet devoid of fruit and vegetables; (ii) the basal diet supplemented with a prescribed amount of cruciferous vegetables (7 g/kg body-weight, termed 1X); (iii) the basal diet supplemented with twice this amount of cruciferous vegetables (14 g/kg body-weight, 2X). MALDI-TOF spectra were collected for each of the diets for the 42 study participants in 2EAT. Peak alignment (coordinated with the EAT data), peak selection and statistical analysis were carried out as described for the EAT spectra. For the 2X cruciferous diet, the analysis identified 24 statistically significant (P < 0.05) peaks that differed from the basal diet. Stratifying the 2X data by genotype, 19 of these 24 were still significant in one genotype or the other. However, independent of these, there were 15 additional peaks that were unique to *GSTM1-null *individuals and 23 additional peaks that were unique to *GSTM1+ *individuals. In addition to diet, these results and those from EAT suggest that the serum peptidome is also being affected by *GSTM1 *genotype in response to cruciferous vegetable intake.

### Combined analysis of diet and genotype in EAT and 2EAT

We focused our analysis on peptides that showed significant and directionally consistent changes with a cruciferous diet. Peaks identified to be consistent in both datasets independent of genotype narrowed the list from 24 in EAT and 2EAT to 4 peaks of interest that shared significant changes and maintained the same trend and appropriate intensity change in both sample populations: 4813, 4905, 9565 and 9812 *m/z *(Table 2).

**Table 2:** Selection of significant peaks affected by diet and/or *GSTM1 *genotype^1^

Peak (*m/z*)		EAT			2EAT					
		
		**1 X Cruciferous**^**1**^			**1 X Cruciferous**^**2**^			**2 X Cruciferous**^**3**^		
		
		ALL	*GSTM1-null*	*GSTM1+*	ALL	*GSTM1-null*	*GSTM1+*	ALL	*GSTM1-null*	*GSTM1+*
4813	**IR**^4^	0.043	0.370	0.005	0.188	0.254	0.140	0.062	0.478	0.062
	
	***p-*value**	0.047*	0.619	0.031*	0.168	0.427	0.240	0.022*	0.664	0.005*

4905	**IR**	1.509	1.214	1.877	1.183	0.920	1.521	1.789	1.436	1.789
	
	***p-*value**	0.020*	0.384	0.022*	0.520	0.823	0.248	0.028*	0.326	0.033*

6500	**IR**	0.870	0.975	0.776	0.823	0.959	0.708	0.527	0.629	0.527
	
	***p-*value**	0.319	0.887	0.242	0.502	0.919	0.391	0.028*	0.248	0.048*

6700	**IR**	0.782	0.933	0.655	0.712	0.878	0.577	0.574	0.876	0.575
	
	***p-*value**	0.123	0.732	0.087	0.276	0.767	0.207	0.076	0.760	0.029*

9565	**IR**	0.744	1.229	0.451	0.745	0.966	0.575	0.569	0.629	0.569
	
	***p-*value**	0.044*	0.267	0.001*	0.064	0.879	0.012*	0.000*	0.038*	0.004*

9812	**IR**	1.218	1.131	1.312	1.167	0.870	1.565	1.494	1.458	1.494
	
	***p-*value**	0.036*	0.297	0.062	0.323	0.529	0.041*	0.011*	0.083	0.058*

^1^1X cruciferous diet (basal diet supplemented with 436 g/day cruciferous vegetables) compared to EAT basal diet (fruit- and vegetable-free).

^2^1X cruciferous diet (basal diet supplemented with 7 g/kg body weight/day cruciferous vegetables) compared to 2EAT basal diet.

^3^2X cruciferous diet (basal diet supplemented with 14 g/kg body weight/day cruciferous vegetables) compared to 2EAT basal diet.

^4^IR denotes the cruciferous vegetable-to-basal diet peak intensity ratio in the unit of square root of estimand of between-subject variance.

* P < 0.05

When we focused the analysis on peaks based on *GSTM1 *genotype, two additional peaks of interest were found to change in a consistent and significant manner in response to cruciferous vegetable intake: 6500 and 6700 *m/z*. Further analysis of significance revealed that the first four peaks shown to be significant by diet were also driven by the *GSTM1+ *participants (Table 2). The intensity ratios of all six peaks show that 4905 and 9812 increase and 4813, 6500, 6500 and 9565 decrease (Table 2).

### Identification of 6700 m/z and 9565 m/z as *GSTM1+ *dependent markers of cruciferous diet

Peaks 6700 m/z and 9565 m/z were selected for identification based on the combination of robust intensity ratios, significance, *GSTM1+ *regulation, and peak height (Figure 1). To attempt to obtain identification for these peaks, the bands of appropriate molecular weights were excised from a gel and proteins of interest were determined via Electrospray Ionization (ESI) MS analysis. A peptide at 6700 *m/z *found to decrease with cruciferous diet yielded ESI spectra consistent with a fragment of TTR, a serum protein responsible for the transport of retinol and the thyroid hormone thyroxine [24,25]. Further, full-length TTR protein has many identified isoforms at approximately 13,400-14,000 Da [26] which would appear as 6,600-7,000 peaks as +2 m/z species.

To test whether the peaks seen in these ranges were TTR, we subjected human sera to C18 HPLC and analyzed fractions via dot blot analysis using an antibody to TTR. Dot blot positive HPLC fractions were then examined via MALDI-TOF MS to identify ones that contained peaks within our range of interest. Fraction 81, eluting at 27% AcN contained peaks at ~6,700 *m/z*. We also spiked purified TTR into the selected HPLC fractions at various concentrations to see if the MS peaks would align at similar *m/z *values. When 50 ug/mL TTR was spiked into fraction 81, there was alignment of peaks in the 6600-7000 *m/z *range including our peak of interest (data not shown). In these spectra there was evidence of the singly charged form of the TTR isoforms at 13,400-14,000 *m/z*, further confirming that several peaks in the 6600-7000 *m/z *range were likely the doubly charged forms of TTR.

**Figure 2:**
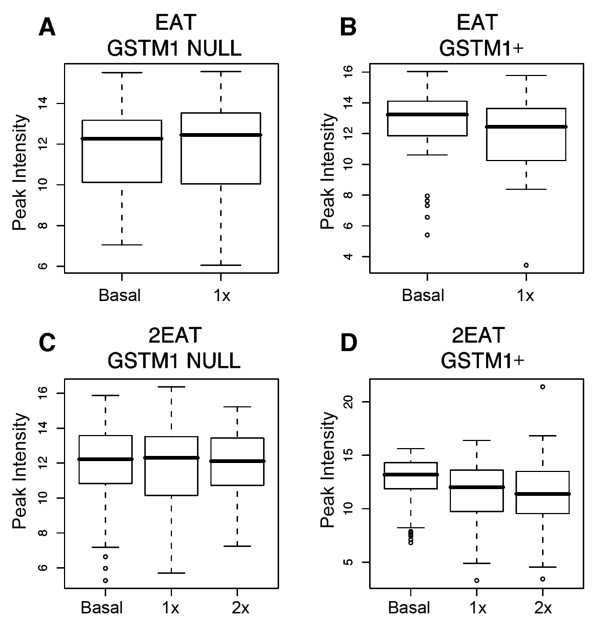
**Box plots comparing peak intensities (normalized to the peak with the highest intensity) of 6700 *m/z *in EAT and 2EAT. **(A) cruciferous vegetable response among *GSTM1-null *individuals in EAT. (B) cruciferous vegetable response among *GSTM1+ *individuals in EAT. (C) cruciferous vegetable response among *GSTM1-null *individuals in 2EAT. (D) Cruciferous vegetable response among *GSTM1+ *individuals in 2EAT.

When we looked at the peak intensity analyses for EAT and 2EAT presented in a box plot, we see lower peak heights for 6700 *m/z *in the *GSTM1 *genotype with increasing amounts of cruciferous intake (Figure 2). Because peak heights may not be directly proportional to the concentration of a protein/peptide, we performed a western blot on a subset of individuals in 2EAT using an antibody to TTR to determine if changes in TTR were correlated with cruciferous vegetable intake. Relative to the basal diet, the change in TTR concentration with feeding the 2X-cruciferous vegetable diet was significantly larger among *GSTM1+ *individuals as compared to *GSTM-null *individuals, 1.24 ± 0.68 and 0.86 ± 0.26 (mean ratio ± SD), respectively (P = 0.001).

**Figure 3:**
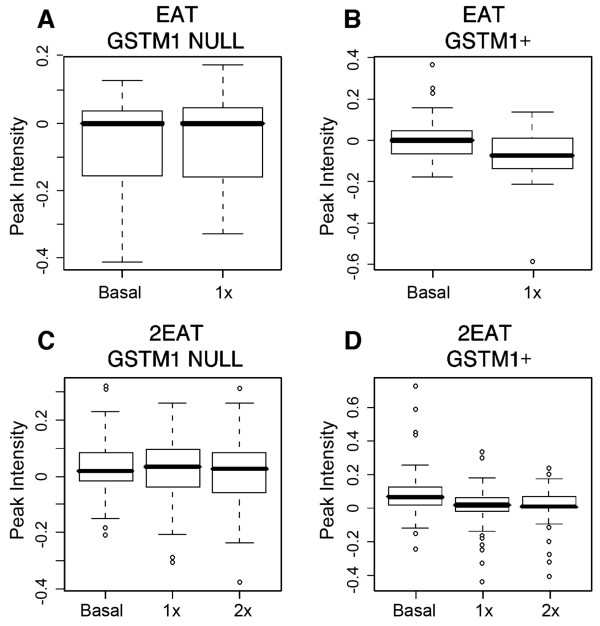
**Box plots comparing peak intensities (normalized to the peak with the highest intensity) of 9565 *m/z *in EAT and 2EAT. **(A) cruciferous vegetable response among *GSTM1-null *individuals in EAT. (B) cruciferous vegetable response among *GSTM1+ *individuals in EAT. (C) cruciferous vegetable response among *GSTM1-null *individuals in 2EAT. (D) Cruciferous vegetable response among *GSTM1+ *individuals in 2EAT.

We further investigated a second peptide at 9565 *m/z *that was found to decrease with all 3 cruciferous diets (Table 2). When we present peak intensity analyses for EAT and 2EAT in a box plot, we see lower peak heights for 9565 *m/z *in the *GSTM1 *genotype with increasing amounts of cruciferous intake (Figure 3).

In order to obtain possible identifications for this peptide, we excised a band at ~9,500 Da from a gel as indicated above and obtained an ESI spectra consistent with ZAG, a serum protein that is known to stimulate lipid degradation in adipocytes [27,28]. Full-length, mature ZAG protein is a glycoprotein of 38,478 Da, so we are likely only detecting a fragment of ZAG.

To further test the relationship of 9565 *m/z *to ZAG, we subjected human sera to C18 HPLC and analyzed fractions via dot blot analysis using an antibody to ZAG. Dot blot positive fractions were then examined via MALDI-TOF MS to identify ones that contained peaks within our range of interest. Two HPLC fractions that contained peaks at ~9500 *m/z *also showed the most signal with the ZAG antibody. Thus, two independent methods of separation and identification were consistent with 9565 *m/z *being a fragment of ZAG.

### Changes in TTR and ZAG levels by *GSTM1 *genotype

To further assess the changes in levels of TTR and ZAG, dot immunoblots of both full sets of EAT and 2EAT serum samples were performed using antibodies to TTR and ZAG. A genotype-specific analysis was carried out on our dot blots to assess the diet differences of TTR and ZAG levels in *GSTM1+ *and *GSTM1-null *individuals.

**Figure 4:**
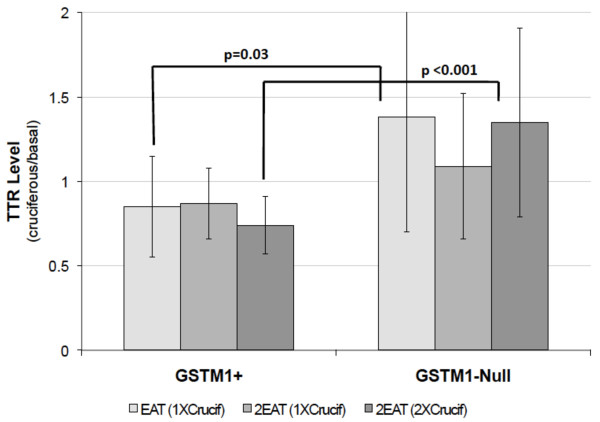
**Diet-genotype regulation of mean TTR level differences via dot blot. **Levels of TTR are calculated as a ratio of cruciferous diet over basal diet for each individual. The values shown are mean ratio ± SD. Samples analyzed include: EAT N = 36 (14 *GSTM1*+ and 22 *GSTM1*-null) and 2EAT N = 42 (20 *GSTM1*+ and 22 *GSTM1*-null).

Among *GSTM1+ *individuals, the mean TTR signal decreased in response to cruciferous vegetable intake as compared to the basal diet (P = 0.030), further supporting the western blot and MALDI-TOF results. The difference between the two genotypes with cruciferous vegetable intake is statistically significant in the 1X cruciferous diet for EAT (P = 0.033) and the 2X cruciferous diet for 2EAT (P < 0.001), but the difference was not significant for the 1X cruciferous diet for 2EAT (P = 0.379). Overall comparison of the two genotypes was significant (P < 0.001) for cruciferous intake across both studies (Figure 4).

We then tested whether TTR could classify participants based on their *GSTM1 *genotype. For this, we used the ratio of the TTR levels in the cruciferous versus basal diets and calculated an ROC curve comparing this ratio's sensitivity and specificity in distinguishing between *GSTM1+ *and *GSTM1*-null. We obtained an area under the curve (AUC) of 0.79 with optimal sensitivity of 73% and specificity of 75% in the 1X cruciferous diet for EAT. For the 2X cruciferous diet for 2EAT, the AUC was 0.90 with optimal sensitivity of 84% and specificity of 100%.

**Figure 5:**
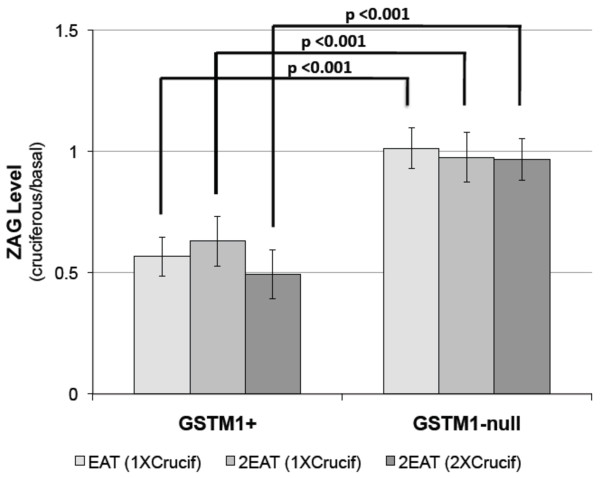
**Diet-genotype regulation of mean TTR level differences via dot blot. **Diet-genotype regulation of mean ZAG level differences via dot blot. Levels of ZAG are calculated as a ratio of cruciferous diet over basal diet for each individual. The values shown are mean ratio ± SD. Samples analyzed include: EAT N = 36 (14 *GSTM1*+ and 22 *GSTM1*-null) and 2EAT N = 42 (20 *GSTM1*+ and 22 *GSTM1*-null).

Among *GSTM1+ *individuals, mean ZAG signal decreased in response to cruciferous vegetable intake as compared to the basal diet (P < 0.001). The difference between the two genotypes with cruciferous vegetable intake is statistically significant in the 1X cruciferous diet for EAT (P < 0.001), the 1X cruciferous diet for 2EAT (P < 0.001), and the 2X cruciferous diet for 2EAT (P < 0.001). Overall comparison of the two genotypes was significant (P < 0.001) for cruciferous intake across both studies (Figure 5).

Sensitivity and specificity were calculated for the ability of ZAG to classify *GSTM1 *genotype when subjects ate a cruciferous diet. ZAG has a sensitivity of 100% and specificity of 100% in all cruciferous diets in both EAT and 2EAT (AUC=1.00 for all diets in both EAT and 2EAT).

## Discussion

We have demonstrated a *GSTM1 *genotype-dependent effect of a cruciferous diet on the proteins found in human serum using two independent feeding studies, EAT and 2EAT. We identified two peaks (6700 *m/z *and 9565 *m/z*) that were affected by the combination of diet and *GSTM1 *genotype and showed consistent trends across diets in both studies as a doubly charged isoform of TTR (6700 *m/z*) and a fragment of ZAG (9565 *m/z*). Using both MALDI-TOF and immunoassays, we demonstrated that both TTR and ZAG decrease with cruciferous vegetable intake among *GSTM1+ *individuals, but not among individuals with the *GSTM1-null *genotype.

TTR is a negative acute-phase protein that decreases in serum with early signs of inflammation. It is a known marker of nutritional status and with a half-life of ~2 days, its concentration closely reflects recent changes in diet [29,30]. TTR has several known biological roles: In cerebrospinal fluid, it is a carrier of the thyroid hormone thyroxine (T4) and retinol. In serum, TTR transports 15% of T4 and retinol through the complex formation with retinol binding protein (RBP). It also binds many aromatic compounds, leading to speculation of its role in removal of toxic compounds [24]. Constituents of cruciferous vegetables at high doses have been shown to suppress the function of the thyroid gland [25]. Thus, although the types and doses of cruciferous vegetables fed in this study were unlikely to influence thyroid function appreciably, one possible explanation of our results is that changes in T4 due to cruciferous vegetable feeding result in less TTR being needed in serum to aid in transport. Another possible explanation is that cruciferous vegetable constituents more directly influence TTR gene expression or TTR protein half-life through other pathways [31].

ZAG is an adipokine and apparently plays multiple roles in the human body, most concerning lipid mobilization from fat stores. Levels of ZAG expression are regulated by glucocorticoids [32]. ZAG is synthesized in the liver and increased levels correlate with lipolysis [33], implying that it may be regulated by fat intake. Decreased levels of ZAG associated with increased cruciferous vegetable intake could relate to adipokine signaling. Another possible explanation is that cruciferous vegetable constituents directly influence gene expression as it has been suggested that ZAG expression is likely mediated by the interaction of several synergistic transcription factors [34]. Investigation into the levels of other adipokines and further analysis of ZAG processing will be necessary to shed light on the biology behind these cruciferous vegetable mediated changes.

These *GSTM1*-genotype-specific responses of TTR and ZAG to cruciferous vegetable intake suggest that differential response to ITC exposure in *GSTM1+ *and *GSTM1*-null individuals may influence the serum peptidome. Whether this genotypic difference is due to a difference in ITC metabolism [12] or another factor remains to be determined. To date, studies have not shown consistent pharmacokinetic differences in ITC handling by *GSTM1 *genotype, but given the rather broad and overlapping substrate specificities of this superfamily of enzymes, other GST isozymes may compensate in part for the lack of *GSTM1* activity [10] or alternative metabolic pathways may substitute for *GSTM1*. By whatever mechanism, several interventions and observational studies suggest a modifying effect of *GSTM1 *genotype on serum GST-α in response to cruciferous vegetable exposure [8,17,35] and as biomarkers of oxidative damage [36]. Based on this previous work and our findings here, it appears that this effect may extend to other serum proteins.

We have also demonstrated that, in the context of a controlled study and systematic data analysis, MALDI-TOF MS is a suitable tool for identifying reproducible biomarkers that respond to diet. In an earlier MALDI-TOF MS analysis of the EAT study, a peptide identified as the B-chain of α2-HS glycoprotein (AHSG) decreased with cruciferous vegetable intake (p = 0.002) [15]. In the current 2EAT single-dose cruciferous diet data this peptide showed a similar trend but without statistical significance (p = 0.197).

The strengths of our study include the controlled feeding study crossover design, the use of two separate datasets to examine the consistency of peak changes across both studies, and the availability of samples from individuals given two doses of cruciferous vegetables in 2EAT for the assessment of dose-response. Limitations of this study include the modest sample sizes, which limited our power to further stratify the data by sex, race, genotype and other possibly confounding factors. Nonetheless, with 78 (36 + 42) participants, we had 80% power (with Type I error probability of 5%) to detect a biomarker difference between the cruciferous and basal diets as small as one-third of its standard deviation. The two feeding studies were slightly different; the feeding period length was 6 days for EAT and 14 days for 2EAT. Further, participants were fed amounts of cruciferous vegetables based on their total body weight in 2EAT, but the same, absolute amount in EAT. In 2EAT, the 2X cruciferous diet also provided doses of cruciferous vegetables that were substantially higher than usual intake reducing generalizability of the results. To reduce inherent variability due to sample preparation and matrix crystallization inconsistencies, samples were run in quintuplicate with multiple preparations for each sample. All samples from each individual in the study were run on the same plate to decrease variability between plates. All plates were run over the course of two consecutive days to limit day-to-day and instrument variability. The peak identification process can be time-consuming and validation can be costly, hence identifying a large number of significant peaks is challenging. Despite these issues, we were able to find peaks with consistent changes in response to cruciferous diet. Further investigation is necessary to establish the mechanism by which crucifers contribute to these protein changes in relation to *GSTM1 *genotype.

## Conclusions

In summary, we demonstrated that the serum peptidome is dependent on *GSTM1 *genotype-diet interactions. Our results support the hypothesis that people of different *GSTM1 *genotypes show changes in serum protein content as a result of metabolic response to diets high in cruciferous vegetables. We identified two notable peaks as TTR and ZAG with high sensitivities and specificities. Particularly for ZAG, these results imply a role in *GSTM1*-regulated dietary biomarkers that should be investigated further. Finally, the study design and the analytic methods utilized here could be applied more generally to intervention and observational studies of genotype-diet interactions.

## Competing interests

The authors (Heather Ann Brauer, Tanya Libby, Breeana L. Mitchell, Lin Li, Chu Chen, Timothy W. Randolph, Yutaka Y. Yasui, Johanna W. Lampe, Paul D. Lampe) declare no conflicts of interest.

## Authors' contributions

JWL, CC, PDL and HAB designed research; HAB, BLM and TL conducted research; HAB, LL, TWR and YYY analyzed data; HAB, PDL and JWL wrote the paper. PDL had primary responsibility for the final content. All authors read and approved the final manuscript.
